# Automatic detection of prosodic boundaries in spontaneous speech

**DOI:** 10.1371/journal.pone.0250969

**Published:** 2021-05-03

**Authors:** Tirza Biron, Daniel Baum, Dominik Freche, Nadav Matalon, Netanel Ehrmann, Eyal Weinreb, David Biron, Elisha Moses

**Affiliations:** 1 Department of Physics of Complex Systems, Weizmann Institute of Science, Rehovot, Israel; 2 Sagol Center for Brain and Mind, Interdisciplinary Center, Herzliya, Israel; 3 Department of Linguistics, The Hebrew University, Jerusalem, Israel; Max-Planck-Institut fur Kognitions- und Neurowissenschaften, GERMANY

## Abstract

Automatic speech recognition (ASR) and natural language processing (NLP) are expected to benefit from an effective, simple, and reliable method to automatically parse conversational speech. The ability to parse conversational speech depends crucially on the ability to identify boundaries between prosodic phrases. This is done naturally by the human ear, yet has proved surprisingly difficult to achieve reliably and simply in an automatic manner. Efforts to date have focused on detecting phrase boundaries using a variety of linguistic and acoustic cues. We propose a method which does not require model training and utilizes two prosodic cues that are based on ASR output. Boundaries are identified using discontinuities in speech rate (pre-boundary lengthening and phrase-initial acceleration) and silent pauses. The resulting phrases preserve syntactic validity, exhibit pitch reset, and compare well with manual tagging of prosodic boundaries. Collectively, our findings support the notion of prosodic phrases that represent coherent patterns across textual and acoustic parameters.

## Introduction

Information in spoken language is conveyed not only through words but concurrently through acoustic cues–fundamental frequency (pitch), intensity (volume), speech rate and rhythm, and timbre, collectively termed Prosody. It is also widely recognized that the distribution of prosodic information throughout the flow of speech is neither uniform nor random (e.g., question/statement boundary tones). Short, often distinctive phrases, which are bounded by prosodic cues, (cf. [1]) convey coherent messages (e.g. [2, 3]) that conveniently avail to the interlocutor a variety of linguistic functions: sentence mode, e.g., assertion vs. question, saliency of information via emphasis, conversation action, discourse function, attitudes and sentiments [4–6]. These units are often referred to as intonational phrases or intonation units (IUs) and although a precise definition is hard to come by, the notion of a well-defined (‘single’) pitch contour is often regarded as a necessary trait [2, 7].

There is no widespread agreement on the nature of intonation units, and even their existence has been contested by some scholars (e.g., [5]). The reasons that we converse using short phrases are unclear. Answers posed in the literature involve the human physiology or aspects of cognitive processes related to the production of vocal output (e.g., [8, 9]). Several lines of evidence support the contribution of the latter: the capacity of our working memory is estimated at 4–7 words (e.g., [10],). Correspondingly, in electroencephalographic (EEG) measurements, event-related potentials show a positive shift in activity at the closure of phrases; this has been accepted as a neural measure for the perception of phrase boundaries (e.g., [11]). Similarly, magnetoencephalographic (MEG) measurements of cortical activity during speech processing revealed a response at an intermediate timescale lying between the syllabic and sentential [12].

Linguistic literature describes a hierarchy of prosodic domains of various lengths, where each element consists of at least one element of the next lower category [13], for example, proposed a hierarchy of six categories: utterance, intonational phrase, phonological phrase, phonological word, foot, and syllable. The intonational phrase–a unit one second and three- to four-word long on average–is central to the study of prosody and interactional linguistics [14]. According to the autosegmental-metrical approach [7], the theoretical basis for the ToBI annotation system, prosodic hierarchy distinguishes five levels of “break indices”. At the higher end of this scale are the (full) intonational phrase boundary and the intermediate (intonational) phrase boundary. IU boundaries, which are the object of our analysis, coincide with both boundary types [5].

A number of qualitative descriptions have been put forward for IUs (see also Dataset section) (e.g., [2, 3] pp. 17–19]). Suggested definitions can be functional (cf. Turn Construction Unit [15]) or acoustic [3]), where the latter will typically focus on a coherent pitch contour and a battery of boundary cues [16]. Autosegmental-metrical theory proposes a hierarchical structure of prosodic constituents, listing their pertinent acoustic patterns (e.g., rise/fall/steep rise/fall etc.). Notably, these patterns are not directly associated with their corresponding discourse functions (e.g., [7], and cf. [17–21] for form/function accounts). According to Fujisaki, too [22], prosodic phrases exist as a locus for patterns extending beyond the syllabic timescale, as well as that of the prosodic word and foot. The related PENTA model suggests that prosodic blocks are defined by their function in discourse and range from the syllabic through the phrasal to the sentential scale [1]. The INTSINT model suggests a system for unit annotation, which is permissive as to specific definitions of prosodic boundaries [23].

IU boundaries are associated in most definitions with a set of typical parameters: slowing down of speech rate at the end of a unit along with acceleration at its beginning, which we denote as discontinuities in speech rate (DSRs); resetting of pitch and/or intensity; a register shift of pitch or intensity; or pausing ([3] Chapter 4P, [14, 16]). Of these, final lengthening together with initial acceleration (DSRs) were identified as particularly salient signals in intonational phrases [16, 24]. Final lengthening is well-documented in conversational American English, as well as in many other languages (see review in [25]). Thus, the intervals between these consecutive DSRs could serve as an automatically measurable proxy for distinct units that bear prosodic-semantic information. There are, of course, other linguistic factors that determine speech rate, such as emphasis [19], syntactic valence [26] along with probability and speech style [27, 28]. It is notable/noteworthy that DSR-based segmentation is sufficiently successful even when these factors are not taken into account. Quantitative support of this notion would enable efficient tagging of prosodic boundaries in recorded conversation. This, in turn, can promote the analysis of non-verbal cues that occur naturally at this time scale of uttering a few words.

All of the above definitions aim to capture the same humanly perceivable phenomenon, yet prosodic units of all scales are difficult to detect automatically. An effective automatic identification of boundaries would extend the power of speech-related applications. Among the advantages of boundary recognition are demonstrated contributions to NLP, possibly as a plug-in in ASR systems. Several conceptual difficulties involved in boundary detection have been shown to improve once a simple and effective automated boundary identification algorithm is made available. The role that human speakers make of boundaries for disambiguation was demonstrated already in [29–31]. There are several examples in the literature which show that, once considered, boundaries reduce error rates for syllable, character, tone, and word recognition (e.g. [32, 33]). Similarly, a prosody-assisted ASR algorithm used ToBI-annotated prosodic boundaries to significantly assist word boundary detection as well as word recognition in English, Spanish and less so in Japanese [34]. Word recognition in scripted English [35], and spontaneous Mandarin [36, 37] was similarly improved by modeling prosodic boundaries. Natural language understanding (NLU) and the resolution of syntactic ambiguities in particular can also be improved when prosodic boundaries are known [37].

Existing automated phrase boundary detection methods often utilize lexical and syntactic cues along with acoustic input (e.g., [38–40]. They usually involve extensive preparation steps such as manual tagging (e.g., [41, 42]) and training a specific, designated model (e.g., [38, 39, 41, 43, 44]). Approaches to speech segmentation based on acoustic signals alone were proposed in [45, 46, 40, 47]. These efforts have been commonly applied to scripted speech (e.g. radio news corpora), where written syntactic conventions prevail and prosody differs significantly from that of spontaneous speech ([38, 39]). Table 1 (see Discussion) lists application of automatic boundary detection in various corpora containing *spontaneous speech*, e.g., the Boston Directions Corpus (BDC) and Columbia Games Corpus (CGC) that include direction-giving tasks and communications relating to specific games, respectively.

**Table 1:** Evaluation of segmentation methods for spontaneous speech.

Source	Dataset	Features	Training	Language	Boundary Detection	F-score	Accuracy
This work	SBC (~28 hrs)	Ac	N	English		0.66	0.86
[47] 2018	C-ORAL-BRASIL (partial, ~9 min)	Ac	Y	Portuguese	F0 + intensity + duration + pause	0.55	0.82
[68] 2017	social media (~6 hrs)	S/L	Y	Chinese + English	Syntax	est. 0.72	
[69] 2016	Proprietary corpus (~5 min)	Ac	N	Spanish + English	F0 + intensity + duration	0.55	est. 0.80
[70] 2016	MGB challenge (BBC TV, ~15 hrs)	S/L+Ac	Y	English	F0 + intensity + duration + syntax + pause	0.63	0.87
[71] 2015	elicited sentences (spontaneous / scripted, ~10 min)	Ac	Y	Romanian	F0 + intensity + duration + pause		0.9
[50] 2013	Valibel Speech Database (spontaneous / scripted, ~6min)	Ac	N	French	F0 + duration + pause	0.93	
[72] 2013	Hungarian BEA (~35 min)	Ac	Y	Hungarian	F0 + intensity		0.78
[73] 2012	CGC (objects game, ~4 hrs)	S/L	Y	English	syntax	0.77	0.89
[73] 2012	Switchboard (partial, ~11 hrs)	S/L	Y	English	syntax	0.43	0.86
[74] 2010	Switchboard (partial, ~2 hrs)	S/L+Ac	Y	English	F0 + intensity + duration + syntax	est. 0.71	est. 0.88
[43] 2009	BDC (spontaneous ~67 min)	Ac per word	Y	English	F0 + intensity + duration	0.81	0.93
[75] 2007	BDC (spontaneous / scripted ~1 hrs)	S/L+Ac	Y	English	F0 + intensity + syntax		0.91 (w/ syntax) 0.83 (w/o syntax)
[76] 2003	Swedish (~25 min)	S/L+Ac	Not specified	Swedish	duration + pause + syntax		0.85
[77] 1998	BDC (spontaneous / scripted ~2 hrs)	Ac	Y	English	F0 + intensity	0.70	0.83

A summary of previous phrase-boundary detection methods that were evaluated using spontaneous speech. Ac = acoustic. S/L = syntactic and/or lexical. Values that were estimated rather than having been explicitly provided are preceded by the qualifier “est.”.

The work presented here describes a method for efficiently identifying a large portion of prosodic boundaries in spontaneous conversation, relying on the output of an ASR system. To verify that the resulting phrases are consistent with human tagging, intervals between consecutive DSRs and/or silent pauses were compared to manually identified IUs. The time course of pitch within these intervals was quantified, showing that this statistical description complements existing qualitative studies of pitch declination (NB, pitch was not used for boundary detection). The data also reveal that intervals between DSRs resemble manually identified IUs from a syntactic point of view, as evident from word frequencies. Since such units are readily perceived and largely agreed upon by humans, these can be deemed sufficient requirements for a good prosodic boundary detection, automatic or manual—regardless of a binding definition of IUs. In addition, the contribution of silent pauses as exclusive boundary cues was quantified. Taken together, our results suggest that identifying prosodic boundaries of the intermediate time scale can promote a better understanding of prosody, as well as significantly enhance and improve the performance of speech processing applications.

## Data set

### The Santa Barbara Corpus

The data set analyzed was the Santa Barbara Corpus of Spoken American English (SBC) [48], published by the linguistics department at UCSB. The corpus consists of a set of 60 audio files that record spontaneous speech of various genres, from multi-party kitchen conversations and couples’ dialogues to child tutoring, guided tours, sermons and university classes. The SBC team recorded audio in two-channel pcm, at 22,050 Hz. The speech files total ~20 hours of audio (7.2GB), representing some 249,000 words in transcription. A transcript (in two formats) accompanies each speech file, where intonation units are time stamped with respect to the audio recording. Here,.trn transcript files were used.

The publishers altered personal identifiers in the transcripts to preserve anonymity. The audio files have been filtered using a digital FIR low-pass filter, with the cut-off frequency set at 400 Hz to make these portions of the recordings unrecognizable. Pitch information is recoverable from the filtered portions, but the amplitude level is reduced.

SBC conversations were transcribed and tagged by students who were trained in a ten-week course on Discourse Transcription at the Department of linguistics of either UCSB (SBC parts I and II, conversations 1–29) or Rice University (SBC parts III and IV, conversations 30–60). About 5 weeks of the course were spent on transcribing intonation units based on the cues described in [3, 14] pp.29-40 and [2]. Students who performed well were hired for the SBC project and provided with additional training [49]. In brief, a unit was defined as “a stretch of speech uttered under a coherent intonation contour. It tends to be marked by cues such as a pause and a shift upward in overall pitch level at its beginning and a lengthening at the final syllable” ([3] Ch 4 pp. 17–19 and cf. [24]). Following the identification of a boundary, the IU was annotated as final or non-final, as defined in [3]. Every conversation was fully segmented and annotated by one student and subsequently fully reviewed by another. Inconsistencies that were not resolved by student reviewers were resolved by an expert (J. Du Bois at USBC for parts I-II or R. Englebretson at Rice University for parts III-IV) [49]. Overall, some 50,000 IUs were manually identified in multi-unit turns, with “turn” being a stretch of speech produced by a speaker before the floor is given to the next speaker. The SBC supplies its user with a transcript, along with markup for boundary tones, laughter, vocalizations, elongation, truncated units, and time codes for each intonation unit.

## Methods

### Measuring speech rate

Speech rate was estimated based on phone durations as obtained from forced alignment (see below). Since phone durations vary within a single word, we estimated speech rate on a time scale of average word duration, thus also improving robustness to noise or minor timing inaccuracies. A time window of 300 ms duration was chosen to approximate the value of the average duration of a word. Varying the window duration in a range of values from 250ms to 500 ms has little or no perceptible effect on the values of the boundary detection and on the accuracy of our methodology.

A speech rate value was then computed for the beginning of every single word, based on the assumption that a phrase does not start mid-word. Specifically, for a word shorter than the window duration, the window could partially include the consecutive word, and for a word longer than the window duration, the window would only partially cover the word. Subsequently, the speech rate value was computed as the mean phone duration averaged over all phones inside this window. Any silence or speech pauses also located inside the window were not included in this averaging.

### Automatic identification of boundaries

Following the estimation of the speech rate, DSRs were detected from the difference (change) between each two consecutive measurements within each turn. Single-unit turns (e.g., “oh yeah” or “u-huh”) were excluded from the procedure. Because there are several prosodic processes that affect speech rate and collude to interfere with an automated DSR-based boundary detection, we developed the following heuristics to reduce the influence of those processes that may falsely constitute DSRs. One example is emphasis, which is particularly difficult to handle for being prominent with respect to its environment. The first heuristic was the use of a threshold (cf. [50]) that was set to 88% of the largest difference in speech rate values of a single turn. Differences higher than this threshold were defined as DSRs and subsequently tagged as boundaries.

We found that the main source of noise in the data is the existence of slowing down in speech that is unrelated to prosodic boundaries. By treating the threshold as an adjustable parameter of the method, the value of 88% proved to be optimal in retaining signal while rejecting noise. It is feasible to improve either one or the other by modifying the value, but an optimum is not apparent or easily found. Thus, the value of 88% incorporates our approximation of the optimum.

The second heuristic was to iterate the DSR detection a second time on those speech stretches between any two consecutive DSRs that were longer than 3 sec. and contained more than 10 words. In this case, the speech rate detection threshold to constitute additional DSRs within such a segment was set to 70% of the largest difference in speech rate values of the stretch.

The third heuristic to specify a boundary was based on the utilization of silent pauses. Timings of silence were derived from the word timings detected by the forced alignment. A silent pause was defined as silence with a duration longer than 300 ms. This value, which is the average duration of a word, corresponds to the optimum for coincidence of manual (i.e., SBC-labelled) and automatic phrases (see Fig 6 below). In the statistical analysis of our detection method, if the same boundary was identified by both a DSR and a silent pause, it was considered to be marked by speech rate. Our method thus defines boundaries as discontinuities in speech rate or as a silent pause, and the interval between two consecutive boundaries is taken as an intonational phrase (see review in [24]).

### Forced alignment

The method we employ relies on the accessibility of the beginning and ending times of each phone. These were obtained using the Kaldi-based forced-alignment software Montreal Forced Aligner (MFA) Version 0.9.0 [51]. The DSR computation time is negligible compared to that of the forced-alignment step which depends on the quality of the dataset and the desired timing accuracy. The MFA was applied by creating a separate acoustic model for each audio file. To avoid invalid alignment originating from overlapping speech, imprecise time codes, or long conversation turns, all turns were split into chunks according to the SBC unit time codes. Subsequently, the MFA was applied to all those chunks originating from a single SBC document. The output of the MFA were the timings of all words as well as phones with the exception of words missing from its dictionary, for which the MFA outputs <unk> without phone timing information. The time resolution of the MFA was 10 ms.

### Measuring pitch

For evaluating the resulting phrases, pitch was measured using Praat phonetics software version 6.0.40, [52], freely available at http://www.praat.org/. The parameters used were Algorithm = autocorrelation, Pitch floor = 75 Hz, Pitch ceiling = 600 Hz. The data were passed through a median filter and the initial distribution of pitch values for each speaker was used to manually identify and correct octave errors and to refine the floor and ceiling parameters. Finally, gaps smaller than 25 ms were interpolated and pitch data were smoothed with a 25 ms Hamming window. To compare contours of different durations we resample all IUs at 40 equally spaced time points, in effect stretching (or contracting) the time axis of short (long) IUs. We refer to this process in the Results section as normalization of the time axis.

### Statistical analysis

Post-processing of automatically obtained IUs was done using custom Matlab scripts [53]. The Mathworks Inc., Natick, MA, USA). Pairwise comparisons of data represented in bar or box plot were done using the Student’s t-test.

## Results

### Speech rate drops ~2-fold at the end of IUs, enabling efficient automatic boundary identification

**Fig 1:**
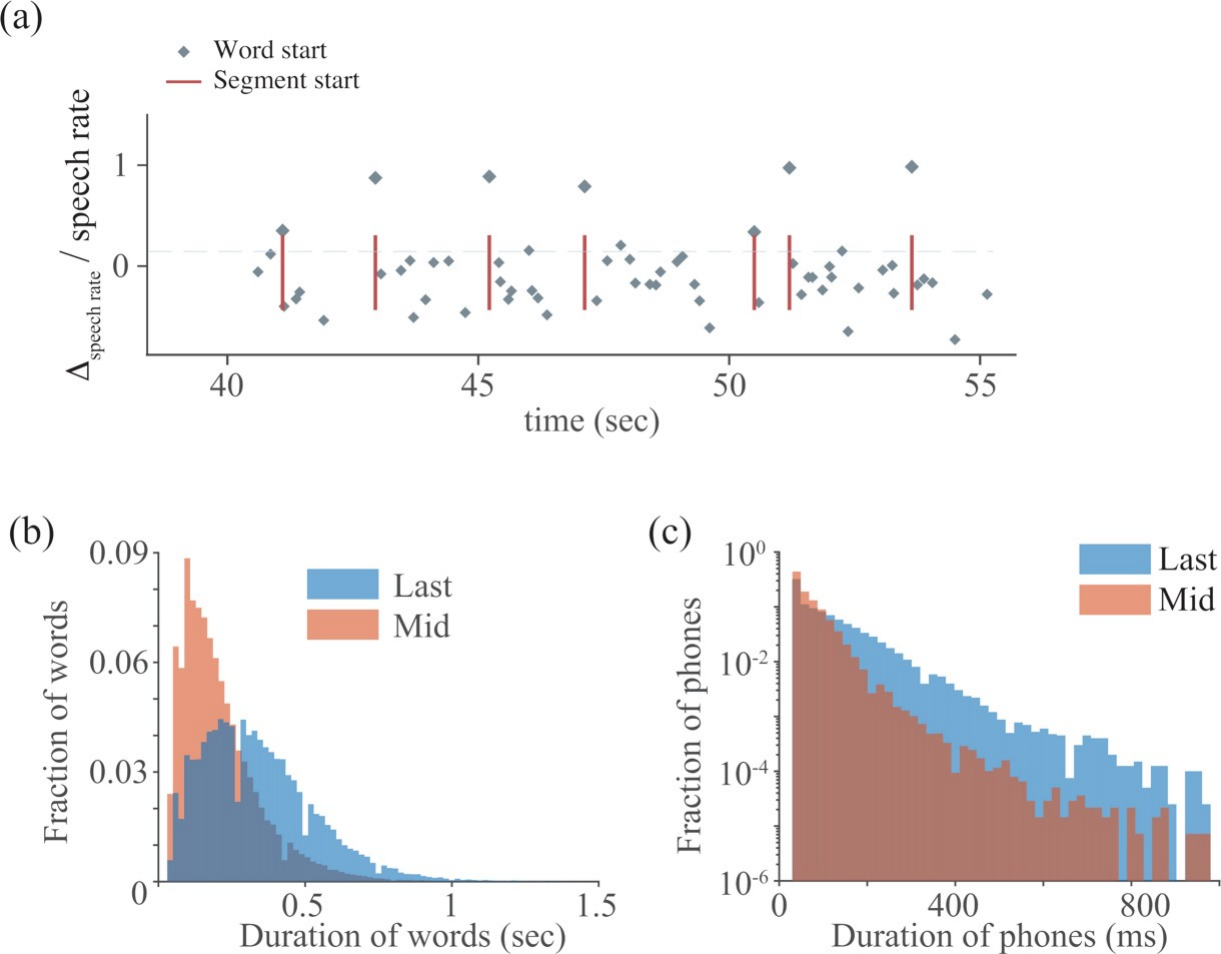
Boundaries of phrases are often signaled by discontinuities in speech rate. (a) An example of boundaries (red lines) of phrases set at word initiations (grey diamonds) that correspond to a peak in relative speech rate. (b) The distributions of durations of middle (i.e., neither first nor last) and of the last words in phrases containing at least 3 but no more 20 words. (c) The distributions of durations of middle (i.e., neither first five nor last 10) and of last phones in phrases containing 3–20 words. N = 60 audio files.

Automatically identifying boundaries without the use of syntax or semantics requires defining prosodic parameters that can be extracted robustly from recorded speech. Examining the manual boundary tagging of the SBC revealed that the relative speech rate (see Methods section) typically peaks at the initiations of IUs (e.g., Fig 1A). Furthermore, the distributions of durations of last words and phones exhibited long tails, suggesting that the slowdown of speech rate is primarily associated with the word terminating the unit (Fig 1B and 1C).

**Fig 2:**
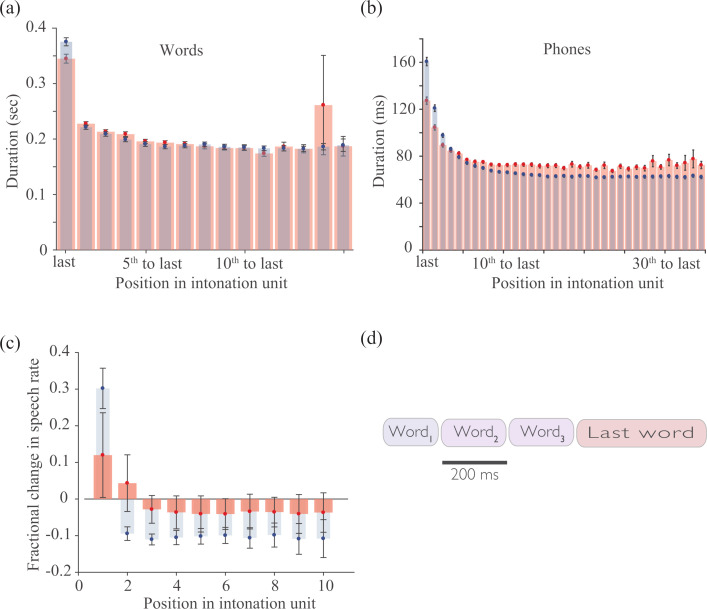
Durations of final words and phones in phrases are extended. (a) Durations of words grouped by their positions, from the last word to the first, in intonation units obtained manually (red) or automatically identified phrases (blue). (b) Same as panel (a), but depicting durations of phones. (c) Relative speech rate is lower at the beginning of manually segmented IUs (red bars). Blue bars confirm the expected trend for in phrases that were automatically identified using speech rate. (d) A sketch of a ‘typical’ IU in the Santa Barbara Corpus. In panels (a, b) phrases containing 2–20 words were considered. In panels (a-c) circles denote mean values and error bars correspond to ± s.e.m. N = 60 audio files.

To demonstrate this, manually identified IUs from the SBC were analyzed as follows: word- and phone durations were averaged by position, ordered from last to first (Fig 2A and 2B). Red bars depict the resulting mean durations for the manually identified units. At the middle of an IU, word and phone durations exhibit narrow distributions with means ± standard deviations (std) of (201±7) ms and (74±2) ms, respectively. In contrast, the mean duration of the last word of an IU was (356±28) ms. This deceleration of speech rate was evident over the last five phones, with the closing phone lasting (118±6) ms.

The durations of the first words in IUs consisting of 3–11 words were mildly (15%) shorter than of words in the middle of IUs. Lengthier IUs were rare and appeared more variable. Single word units comprise approximately 20% of all within-turn IUs; their durations were similar to those of final words and phones as opposed to initial ones. To control for averaging artifacts, these trends were confirmed by measuring durations separately for IUs comprised of equal numbers of words (ranging from 3–20). Together, these data suggest that the speech rate typically changes at the boundaries of manually identified IUs, predominantly due to a significant slowdown at the last word.

For the sake of completeness, a similar analysis was performed on automatically identified boundaries (Fig 2A and 2B, blue bars). In addition, the fractional changes in speech rate for manual and automatic intervals are depicted in Fig 2C. Automatic detection was based on a local measurement of speech rate, defined by the multiplicative inverse of the duration of phones within a fixed temporal window (300 ms–comparable to the mean duration of a word). The duration of the window did not require fine-tuning: values between 250 and 500 ms produced similar results. However, performance strongly depended upon aligning the initial time point of each temporal window with the onset of a word, as determined by forced alignment. This strict requirement led to the data-driven assumption of the method that a boundary cannot occur within a word.

The automatic detection proceeds by identifying both the time points at which speech rate increases abruptly beyond a threshold value and silent pauses of minimal duration. Boundaries were defined as time points at which at least one of these two conditions was satisfied. For the purpose of comparisons with manual IUs, the termination of an automatically identified phrase was set to the termination of its last word. As expected, IUs thus found accentuated the slowdown in speech rate towards their ends: the durations of the last and middle words (mean ± std) were (370±23) and (199±6) ms, respectively. The durations of last versus middle phones were (127±11) and (76±1) ms, respectively (Fig 2A and 2C, blue bars). Thus, Fig 2D depicts a ‘typical’ IU by reflecting the trends observed between manually or automatically identified boundaries.

The method achieves, with modest computing power and a single pass, a rate of analyzing one hour of audio data in about 30 sec. Runtime is mostly dedicated to i/o. Preparation of the data included only a standard forced alignment step (i.e., overall runtime should include that of an engine, roughly 1 minute per 1 minute of recorded speech). In summary, a slowdown/acceleration at the last/initial word of units in American English spontaneous speech and a robust measure of local speech rate enable efficient automatic identification of boundaries.

### Intervals between consecutive DSRs are largely consistent with manual tagging

To further characterize the automatically identified phrases, their durations (in time), lengths (number of words), and boundary time-points were compared to manually segmented units. Although, strictly speaking, there is no universally accepted definition of an IU, all available definitions aim to capture the same humanly perceivable phenomenon (e.g., [11, 12]). Different definitions thus typically agree on the bulk of manually identified boundaries. Similarly, automatic phrase boundary detection applied to a large dataset should produce overall distributions that are consistent with manual tagging.

The manual and automatic boundary tagging of multi-unit turns (i.e., turns containing more than one line according to the SBC markup) yielded 50,324 and 47,640 IUs, respectively. Of these, 31,960 boundaries (67%) coincided between the two methods; some SBC files gave better results than others (78% vs. 55%; chance 25%). This can be compared to the typical agreement between human taggers in ToBI studies ~90% in read, scripted speech (e.g. [54]), and, more directly, to the 78% agreement of boundary identification in spontaneous speech [8]. Nevertheless, DSR boundaries capture a large fraction of units as compared to the best available approximation of a ground truth.

**Fig 3:**
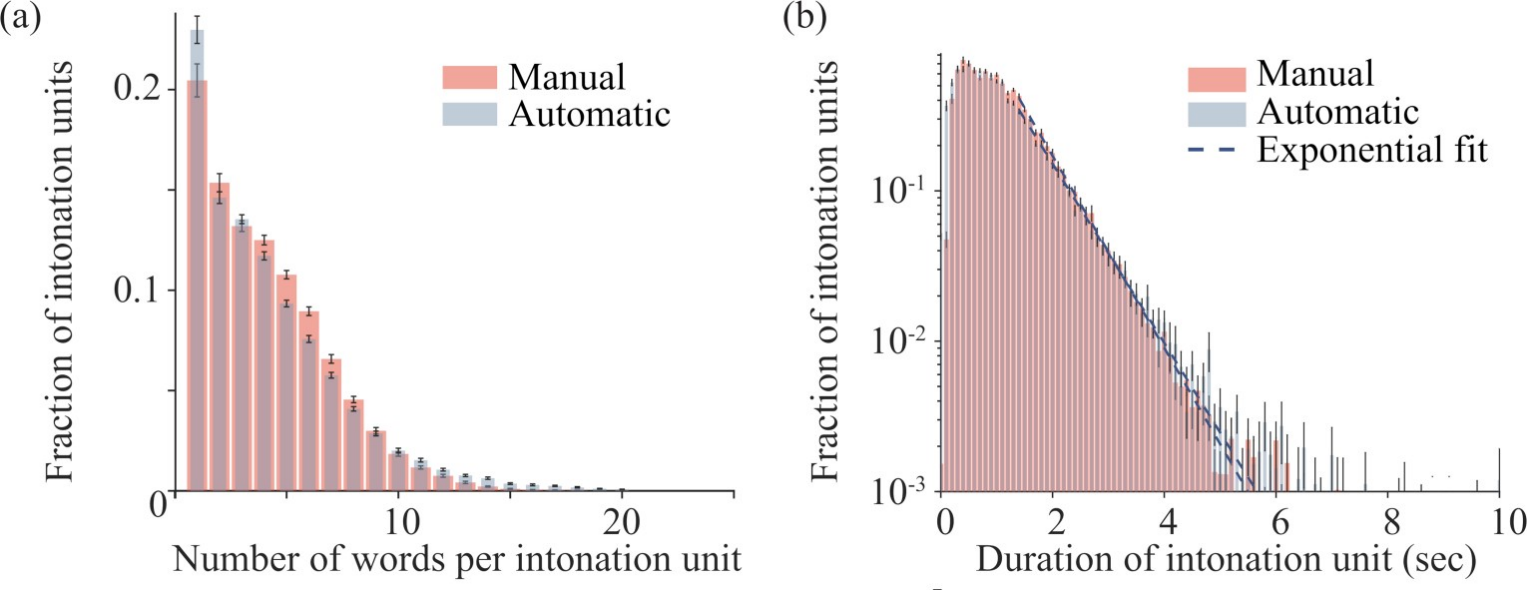
Automatic and manual taggings yield similar distributions of durations and length. (a) The distributions of the number of words per IU for automatic (blue) and manual (red) tagging. Mean durations were (4.26±0.08) and (4.10±0.07) words, respectively. (b) The distributions of durations of IUs for automatic (blue) and manual (red) tagging. Mean durations: (1.08±0.03) sec and (1.14±0.03) sec, respectively. Dashed lines denote exponential fits to the tails of the distributions; time constants: t = 0.73 sec and 0.68 sec, respectively; goodness of fit: R2 = 0.993 and 0.998, respectively. In both panels, the calculation was performed for each audio file individually: Error bars correspond to ± s.e.m. N = 60 audio files.

The average numbers of words (mean ± s.e.m) in manually and automatically obtained phrases were (4.10±0.07) and (4.26±0.08) words, respectively (Fig 3A). While 1% of the manually identified IUs contained more than 12 words, 3% of the automatically identified phrases surpassed this length. In part, this difference may be due to isorhythmic speech, for which the fixed threshold may be too coarse. However, these outliers have only a small effect on the measured distributions.

Fig 3B depicts the distributions of durations of IUs for each boundary detection method. Durations (mean ± standard deviation) of manually and automatically obtained units were 1.0±0.8 sec and 1.1±0.9 sec, respectively. Both distributions exhibit a peak near zero and an exponential tail, which are the characteristic structure of the Poisson distribution, i.e. one that is formed by a random process. We conjecture that this may be because the statistics of interruptions is determined by a diverse array of factors. For example, the exponential decay may reflect the fact that longer IUs can be terminated by a battery of physiological and cognitive factors such as breathing requirements or self-repair.

We observe that the automatic tagging yields a greater number of the overall rare long IUs compared to the manual method. Some of these differences represent systematic (potentially correctable) errors. Other cases may not represent errors in the automated method at all; the differences result from the occasional tendency of the SBC’s human taggers to prefer syntax over prosody when encountering deviations from conventions of written syntax–a frequent phenomenon in conversation (e.g. when a speaker retains the turn of the conversation by using a final “and”, “so” or”that”).

### Intervals between consecutive DSRs mirror pitch dynamics of manually identified IUs

**Fig 4:**
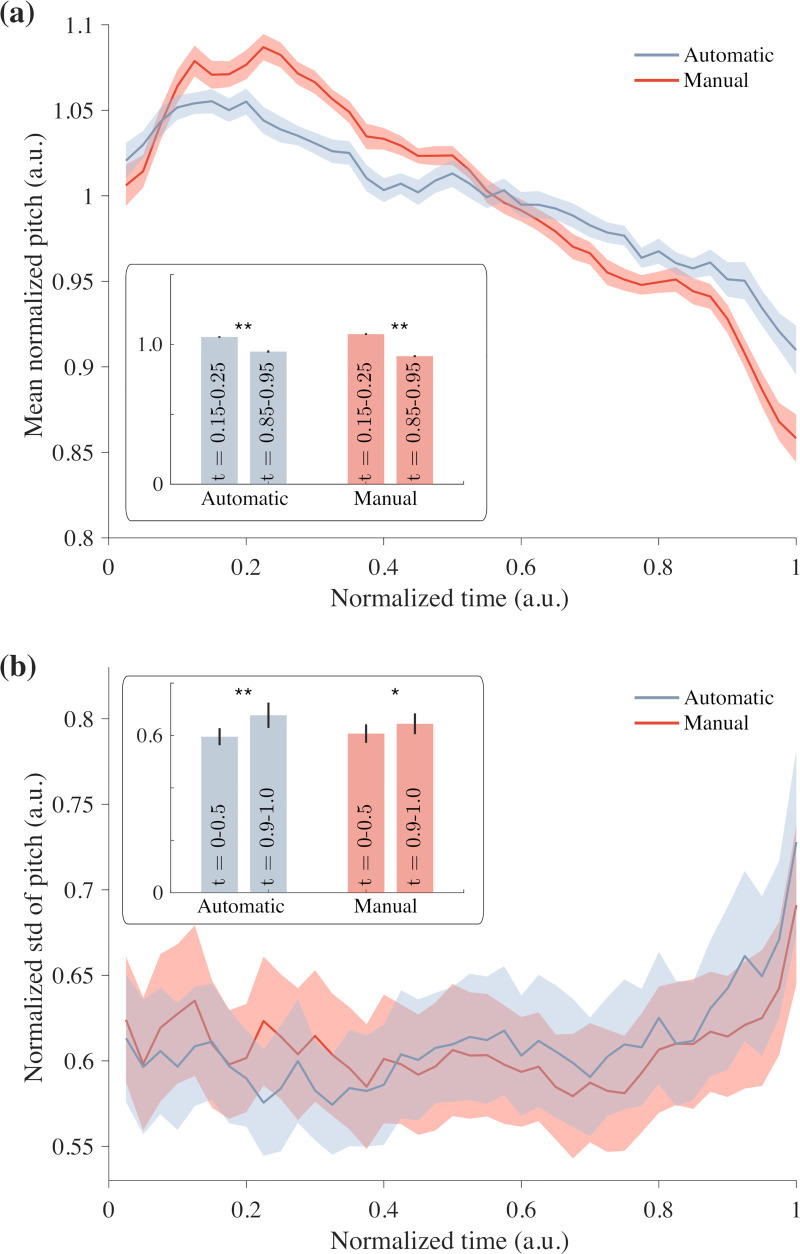
Automatic and manual tagging exhibit pitch reset. (a) Mean normalized pitch as a function of normalized time exhibits a peak near the initiation of a phrase. Blue: automatic phrase boundary detection. Red: manual boundary detection. Inset: the average pitch at time intervals t = 0.15–0.25 (beginning) and t = 0.85–0.95 (end). Asterisks denote that the average was significantly higher at the beginning: p = 2x10^-9^ (automatic) and p = 10^−16^ (manual). (b) The standard deviation of the pitch as a function of normalized time is higher near termination of phrases. Blue: automatic boundary detection. Red: manual boundary detection. Inset: the average standard deviation (STD) at normalized time intervals t = 0–0.5 (first half) and t = 0.9–1 (end). Asterisks denote that the STD was significantly higher at the end: p = 0.0004 (automatic) and p = 0.017 (manual). In both panels, the calculation was performed for each audio file individually: N = 60 audio files. Lines and shaded areas represent mean and ± s.e.m., respectively.

Pitch reset, i.e., the resetting of pitch following its decline over the duration of a unit, is accepted as a common hallmark of IUs (e.g., [3, 4, 55, 56]). Therefore, although not employed for automatic boundary detection, pitch contours between consecutive boundaries should mirror the dynamics of pitch of manual IUs. As expected, pitch reset is readily apparent in SBC manually identified units (Fig 4A, red curve). To test that automatically identified phrases exhibit similar dynamics, pitch was extracted *post factum* using the Praat software, normalized, and plotted as a function of normalized time (Fig 4A, blue curve). The early peaks seen in both curves are caused by pitch resetting: the pitch declines, on average, by 10–20% throughout an IU. In both data sets, manual and automatic boundary detection, peak pitch was reached at normalized time t = 0.2. Given the mean duration of first words of IUs (Fig 2) and the distribution of IU durations (see below), the timing of peak pitch would typically correspond to the second word of the IU.

In the existing literature pitch is also hypothesized to exhibit heightened variability at the end of an IU, due for example to boundary tones ([4, 55, 57, 58]). To test this, fluctuations in pitch as a function of normalized time were plotted for both boundary detection methods. Indeed, the variance during the last 10% of an IU was significantly higher than during the first half of the IU, although this measurement itself was highly variable. On average, the variance at the end of an IU rose by 15% (manual boundary detection) or 20% (automatic boundary detection) with p = 0.02 and 0.001, respectively (Fig 4B). The time interval that corresponded to this change in variability was comparable to the duration of the last phone in the IU.

To summarize, although the boundary detection itself did not make use of pitch data in any way, and although the recordings varied in speakers, genre and communicative purpose, a consistent and clear pitch reset was observed. As expected, randomly segmenting speech into intervals of about one second (the mean duration of a phrase) and then averaging over them exhibited no such decline in pitch. We conclude that measurements of pitch reset and of pitch variability at the closure of phrases support the notion of similarity between the automatic and manual boundary detection.

### Words likely to immediately follow a DSR mirror the words frequently opening manually tagged IUs

An automatic method that ignores syntax should nevertheless preserve known relations between syntax and prosody, similar to how they are preserved in the manual boundary detection. To characterize the syntactic structure of automatically identified phrases, the frequency and identity of the most prevalent words were examined as a function of their positions. The most frequent words in each position are listed in Table 2. Analyzing the manual and automatic boundary detections separately allowed to validate the latter, and also highlighted some characteristics of conversational language in the SBC.

**Table 2:** 

**Rank**	**Automatic**			
	**pos. 1**	**pos. 2**	**pos. 3**	**pos. 4**
**1**	and	I	the	the
**2**	I	the	I	to
**3**	you	you	you	a
**4**	the	know	a	I
**5**	but	it	to	you
**6**	that	was	that	that
**7**	<unk>	and	was	it
**8**	yeah	that	it	of
**9**	so	a	and	and
**10**	well	he	know	in
**Rank**	**Manual**			
	**pos. 1**	**pos. 2**	**pos. 3**	**pos. 4**
**1**	and	I	the	the
**2**	I	know	a	to
**3**	you	the	to	a
**4**	but	you	you	that
**5**	yeah	was	was	it
**6**	so	a	I	of
**7**	the	he	it	you
**8**	well	it	that	in
**9**	that	they	know	like
**10**	oh	don’t	have	was

Each column lists the most frequent words, by order, for each of the first four positions in the phrases. Blue: identical words occupying the same position in manual and automatic boundary detection. Red: verbs.

**Fig 5:**
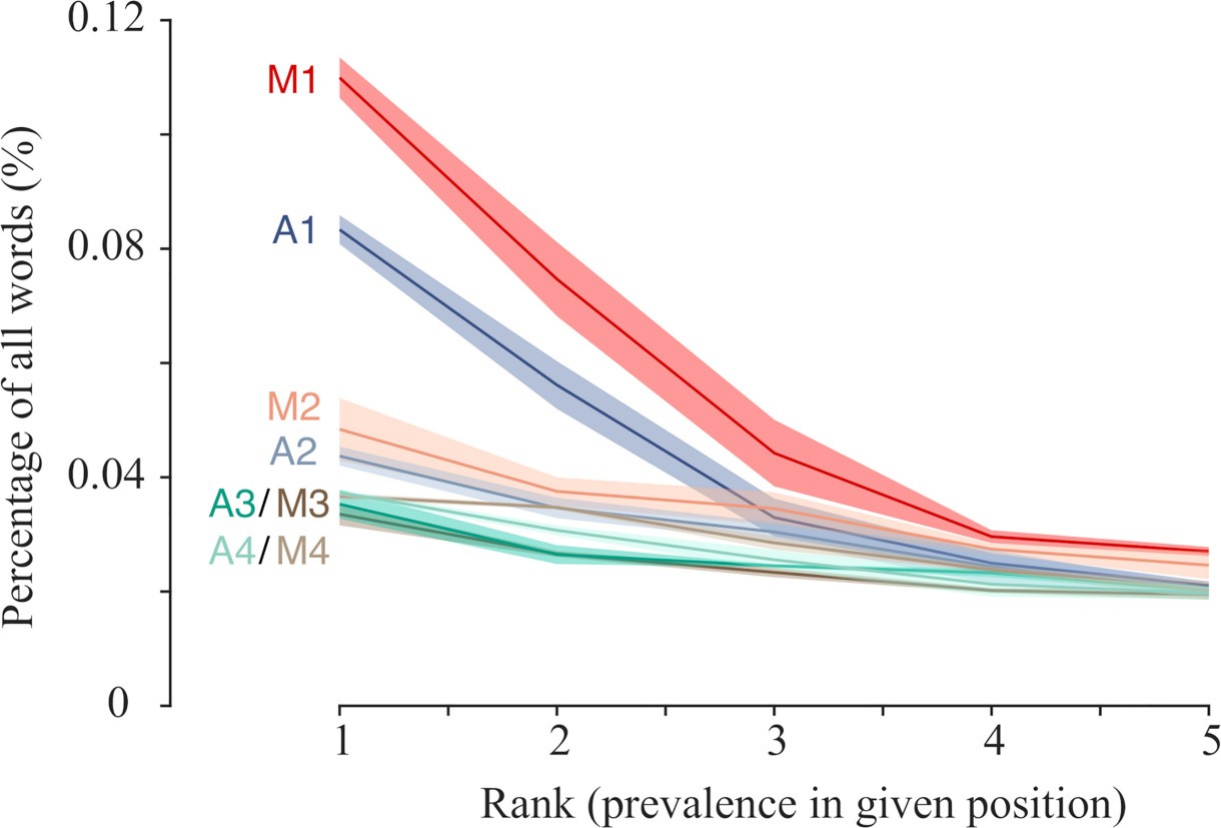
Frequent words are over-represented at beginnings of automatically identified phrases. For each of the first four positions of the phrases and for each of the five most popular words found in that position, the probability to appear at that particular position was calculated. This was done by dividing the number of times that a word appears at a particular position by the total number of times this position was found in the dataset. To evaluate the errors, a calculation was performed for each of the N = 3 groups (of 20 audio files each) individually. Lines and shaded areas represent mean and ± s.e.m., respectively. M1-M4: curves based on manual boundary detection. A1-A4: curves based on automatic boundary detection.

A salient finding was the predominance of “and” in the first position. Coordination (typically “and” and less frequently “but” or “so”) is known to appear in spoken English three times more frequently than in correspondence and nine times more frequently than in academic writing [59]. The frequent appearance of the pronouns “I” and “you” in the first and second positions is a further indicator of the syntax of conversational language. For instance, the pronoun ‘I’ accounted for 6% and 8% of all phrase-initial position in automatic and manual boundary detection, respectively. The appearance of verbs in the second and third positions is yet another such indicator [60]. The statistics of the phrase-opening positions are known to exhibit unique properties, with a specific group of morphological classes–pronouns, subordinates and connectors—appearing more frequently than any word in any other position [61, 62]. Fig 5 shows the frequencies of occurrence of the words most likely to occur at positions 1–4 of IUs, normalized by the total number of words used at that position. Words were then ranked from the most to the least used and the percentage data for the five most frequent words in each position were plotted. Errors were estimated by dividing the data into three groups, each consisting of 20 recordings of comparable durations.

Both methods show a significantly higher peak at the initial position as compared to positions further down the IU. For instance, the most frequent word in the third or fourth position is ‘the’, and it constitutes about 2–3% of all words appearing in in each of the positions 2, 3 and 4. In contrast, ‘and’ was found in 11% (manual) or 8% (automatic) of all phrase-initial positions, or about three times more frequently than ‘the’, as indicated by the leftmost points in the respective curves. Words further along the IU exhibited behavior that is similar to the fourth position. To rule out the possibility that mostly IUs of length one or two words dominate this trend, we verified that it persisted even when only IUs longer than three words are considered. IU lengths longer than two or four words exhibited these trends as well. Notably, these analyses are consistent across the division into three groups of audio recordings, performed for statistical control. Taken together, consistency with manual boundary detection and reproducibility between data sets suggest that this automatic phrase boundary detection yields phrases that mirror the syntax of manually identified IUs.

### Boundaries identified exclusively by a silent pause are infrequent in spontaneous speech

**Fig 6:**
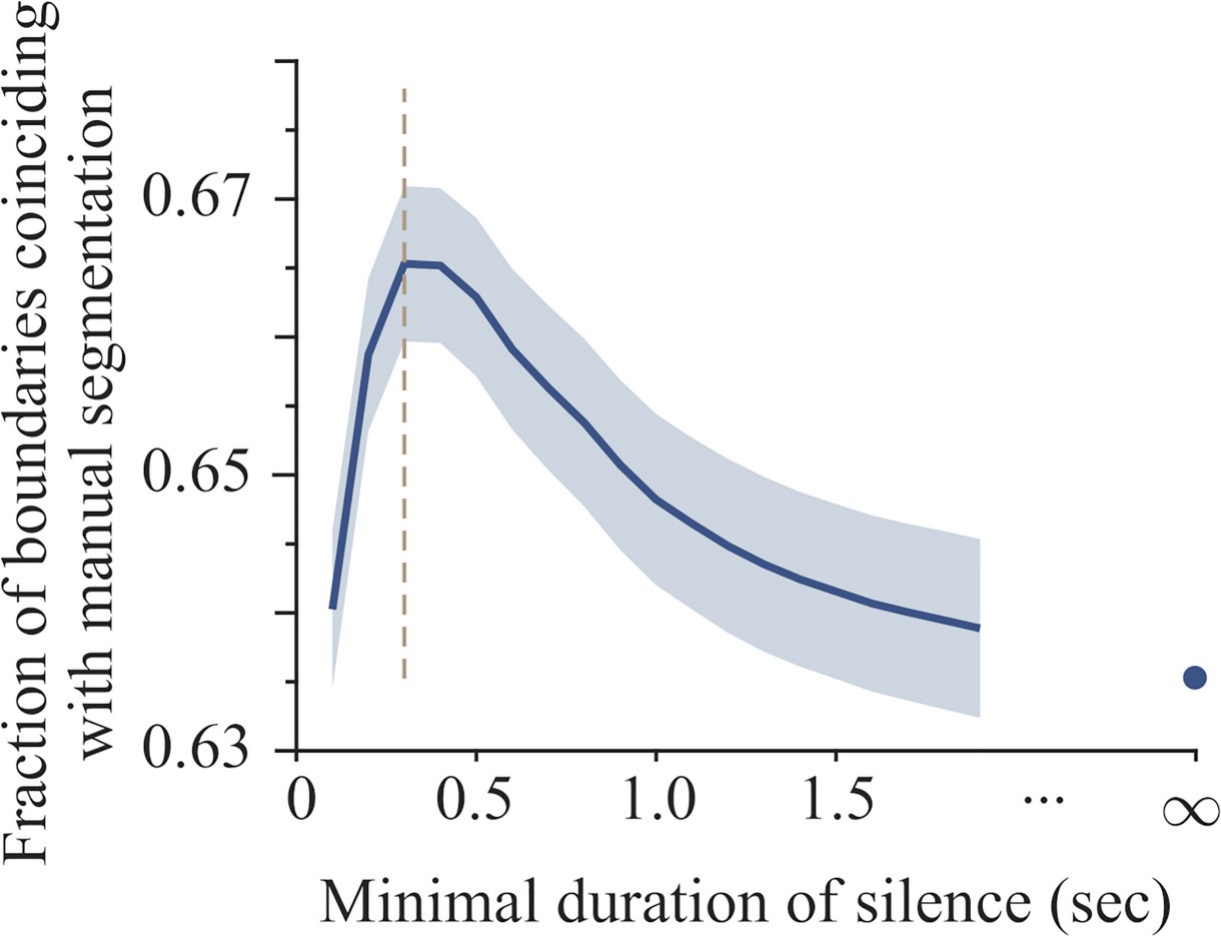
Pauses comparable to (or longer than) the duration of a word mark boundaries. For each threshold value of the minimal duration of meaningful pauses, the phrase boundaries were identified using criteria of both speech rate and of pauses longer than the threshold value. For each resulting boundary detection, the precision was calculated as compared to manual boundary detection. Precision as a function of the threshold values peaked at 300 ms (denoted by vertical dashed line)–a value comparable to the mean duration of a word. The calculation was performed for each audio file individually: N = 60 audio files. Lines and shaded areas represent mean and ± s.e.m., respectively. The point at an infinite threshold represents the mean precision obtained when pauses were not used, showing that including pauses increased the agreement between automatic and manual boundary detection by 2.5%.

It has been hypothesized that pauses are infrequently used as the only marker of an IU boundary [3], i.e. with no concurrent change in speech rate (cf. [14]). If so, ignoring pauses should have only a limited effect on the resulting phrase boundary detection. To quantify this hypothesis, the automated boundary detector was used to count boundaries that are marked exclusively by a pause, i.e., not by a change in speech rate. Next, the minimal duration Δ_min_ of a pause that was considered as defining a boundary was varied in the range of 100–1900 ms. For automatic boundary detection corresponding to each value of Δ_min_, the fraction of IU boundaries that were also identified manually, i.e., the precision as compared to the manual tagging, was measured (Fig 6).

The peak value was reached at Δ_min_ = 300–400 ms, where pauses increased precision by 2.5%. Irrespective of manual boundary detection, (80±1)% of the boundaries identified with Δ_min_ = 300 ms were also identified automatically when pauses were not used as a marker for boundaries at all. Here, the standard error of the mean was calculated by treating each audio file as an independent measurement (N = 60). Interestingly, 300–400 ms is approximately the duration of a last word and is larger than the typical word durations at early or mid IUs. Combined, these results suggest that silent pauses can mark IU boundaries even in the absence of significant changes in speech rate; however, this occurs infrequently within a turn in spontaneous speech.

## Discussion

### Previously reported boundary detection methods

Computationally efficient boundary detection of spontaneous speech is a long-standing problem (e.g., [43, 63]). Often, syntactic and lexical models are combined with acoustic cues, and machine learning is used for classification. Such methods can require extensive preparation such as manual tagging or model training (e.g. [38–41, 43, 64, 65]). Moreover, many previously reported methods have been applied to scripted speech. When reading from a script, prosodic and syntactic boundaries coincide, written conventions being more pervasive and disfluencies rare. In addition, prosody naturally differs overall between scripted and spontaneous speech. Boundary detection in spontaneous conversations is thus a distinct problem.

The sizes and domain specificity of reported datasets should also be considered. Some corpora are small, repetitive, or focused on a specific task. For instance, the BURNC contains a limited number of stories repeated by several speakers. The Boston Directions Corpus (BDC) and Columbia Games Corpus (CGC) contain brief direction-giving tasks and communications relating to specific games, respectively. In such cases, training and testing data may be correlated, models may learn qualities unique to the dataset, and performance may not be preserved more generally.

Different metrics are reported for evaluation of boundary identification, mostly derived from the true positive (TP), false negative (FN), false positive (FP), and true negative (TN) values of the classifier vis-à-vis the reference. Precision (p), recall (r), F-score (f), and accuracy (ac) are defined as TP/(TP+FP), TP/(TP+FN), 2*p*r/(p+r), and (TP+TN) /(TP+FP+FN+TN), respectively [66]. While a one-to-one correspondence between r and/or p and ac does not exist, for a well characterized dataset some missing values can be estimated. It is possible to define the agreement between the manual and automatic boundary detection using Cohen’s kappa [67]. Applying Cohen’s Kappa test to our results yields κ = 0.79, which may be compared to κ = 0.58 as in [8] assessed for inter-annotator agreement between non-experts.

Table 1 compiles thirteen papers that have developed boundary detection methods, and compares their methodologies and results. Seven of these used datasets longer than one hour. Of those only the Switchboard and the BBC datasets are as varied and rich in prosodic components as the SBC. The Table summarizes evaluation metrics of previous boundary identification methods for spontaneous speech. Values that were estimated rather than having been explicitly provided are preceded by the qualifier “est”. In addition, the table notes the type of features used (solely acoustic and/or syntactic/lexical) and whether the method required model training.

There is clearly a large variety in approaches, datasets and the results seem to correspond to these. Seven of the 14 methods use text or syntax, and thus require transcription. Learning models do well, yet require high amounts of resources, in the form of initially annotated datasets, presumably supplied by a human annotator. This is especially potent in dealing with short datasets, where the data typically includes less variability in the prosodic forms that it contains. Without learning, models preform less optimally, even on short data sets. An outlier to this trend is [50], which performs well (F = 0.93) despite using only acoustic resources, and not using a learning model, albeit on a short dataset. Presumably the reason for this is that the authors manually assigned annotation for the syllables in the text, and furthermore removed syllables that were characterized as hesitations. This reduces noise and variability in the speech rate of the final unit, allowing for high precision.

Our results can be compared to those obtained [70] with the BBC data (F = 0.63), and the values for the F-score with our methodology (F = 0.66) are very similar. Another comparable result is that of [73] (F = 0.43) using the Switchboard data. The highest F-score using a varied dataset with over one hour was obtained in [74] for the Switchboard (F = 0.71), with a methodology that relies on syntax coupled with acoustics, using a learning model.

Training a model of boundaries in spontaneous speech using syntactic, lexical, and acoustic inputs can be effective (F-score 0.69) [78]. Boundaries in spontaneous speech were most successfully identified when the training/testing dataset was domain-specific, such as the direction-giving tasks in the BDC (F-score 0.81) [43] or the positioning instructions of the Objects game in the CGC (F-score 0.77) [73]. Provided extensive training, language models (independent of prosody) can identify boundaries with F-scores of 0.70–0.75 [68, 79]. Finally, language models and acoustic cues were successfully combined to identify full stops in spontaneous speech [41, 79, 80]. However, as compared to phrases, sentences often terminate more prominently and the smaller sentence to word ratio (large number of TN) bolsters the accuracy metric. Thus, the method proposed here (F-score 0.65, no training, large and variable corpus of conversations, easily adjustable parameters) compares well with previous methods for segmenting spontaneous speech.

Boundary detection is expected to be influenced by genre, and Table 3 shows this effect on our data set when it is divided into two groups: 48 conversation files and 12 audience-oriented files. A chi-square test of independence gives χ2 (3, N = 37,118) = 305.31, p < .001, i.e., boundary detection in audience-oriented talk is more successful. This suggests that parameter values which were found optimal for the entire data set (see Methods section) may be further optimized per genre, thus leading to better detection.

**Table 3:** Detection rate in conversational vs audience-oriented files.

	Genre		
Detection	Conversational	Audience-oriented	Total
**True Positives**	16171 (16479)	4973 (4665)	21144
**True Negatives**	100664 (101491)	29560 (28733)	130224
**False Positives**	13050 (12350)	2797 (3497)	15847
**False Negatives**	12884 (12449)	3090 (3525)	15974
**Total**	142769	40420	183189

The table shows observed values and expected values (in parenthesis). Words that are followed by speaker change were left out.

In summary, the main advantage of our methodology is its simplicity and its low demand in terms of resources, while remaining efficient and universal. The focus on using a local speech rate criterion enables accommodating the different speech styles and circumstances of communication.

### Pre-boundary lengthening/acceleration (DSRs) and pauses suffice to define boundaries in conversational English

IUs are hypothesized to be a universal linguistic phenomenon (e.g. [81]), with links to speech production constraints, despite the ongoing debate regarding their precise definition. They are demonstrably identified by human perception [8], but present an ongoing challenge for quantitative modeling and therefore for automatic detection (cf. [24, 82]). Suggestions such as the Fujisaki model [22], INTSINT [23] or PENTA [1] define IUs implicitly while the functionalist and Autosegmental-metrical models are explicit [3, 7].

The prevalence of pre-boundary lengthening in conversational English is supported by our measured coincidence of 0.66 F-score and 0.84 accuracy of manually- and automatically-tagged boundaries, a figure which should be compared with the ~80% inter-human agreement in spontaneous speech. Thus, although other cues may improve boundary detection, we posit that DSRs and silent pauses provide in and of themselves a quantitative definition of a usefully large portion of boundaries in English.

In our method, detection is expected to be affected by prosodic hierarchy, since the higher a unit is located in the prosodic hierarchy, the more extensively marked is its prosodic boundary (a well attested phenomenon, e.g., [5, 21]). This may introduce some bias in our results, such that within the total count, final boundaries will be over-represented. Indeed, the recall for detection of final units is 69.4%, whereas for non-final units it is 51.3% (IUs followed by speaker change were not considered). A chi-square test of independence (using the Yates correction for 2x2 contingency tables) gives χ2 (1, N = 37,118) = 1064.55, p < .001. That is, final/non-final populations are significantly different. This is comparable to the results reported in the literature and evaluated in Table 1: a study on data with duration of about 9 minutes [47], the recall for final units was 80% while for non-final units it was limited to about 40%. Similarly, [50] studied about 12 minutes and yielded a detection recall 74% for final boundaries and of 44% for non-final unit boundaries. In their study of about 25 minutes of data from the BDC, [76] report a recall of 49% for final and 42% for non-final boundaries.

### Measurements of speech rate

Our use of speech rate as the main determinant for phrase boundaries has the advantages of being expedient, easy to calculate and efficient in the context of automatic speech recognition applications. Just as important is the fact that our speech rate measurement is relative to its near vicinity and thus sensitive to contextual changes. By addressing speech rate differences to find the largest changes, our method finds boundaries even in relatively monotonous speech or in very rapid sequences. As noted, variety of automatic speech recognition and generation applications analyzes speech rate to improve their performance (e.g., [32, 83]).

One limitation of our method is that the speech rate measurement requires ASR output/forced alignment, i.e., the existence of a transcript. While speech rate can be extracted directly from a soundwave through automatic syllable count estimation, these methods are currently not sufficiently accurate for our purposes (e.g., [84] and cf. [85]).

It is significant that in our hands aligning the measurement of speech rate with the onset of words proved essential, indicating that there is an effective exclusion of boundaries from the middle of a word. Lexical words may not be acoustically determined, but they do contribute to a cleaner boundary signal. While looking for boundaries strictly at the onset of every word may be an imperfect heuristic, it can be modified using pitch-templates and additional cues.

Fluctuations in speech rate which do not stem from boundary signaling (e.g. emphasis) may cause errors in boundary detection. The existence of a cutoff threshold value (of 88%) serves to alleviate many of these errors. In the example of emphasis, the signal produced will be slightly lower than what a prosodic boundary will produce, and the threshold effectively overrides many of these instances.

### Pitch decline/reset

We and others have found that pitch, as compared to speech rate, provides an overall weaker signal for boundaries of IUs [46]. A decline in pitch along the IU is a salient phenomenon that we readily detect by averaging over phrases in our sizeable dataset. However, individual pitch contours are too variable to be trusted as a signal [24]. Thus, our method does not include pitch as a criterion for finding IU boundaries.

Once boundaries are detected using speech rate, we could use the phenomenon of pitch reset at the boundary to verify our method and provide a ‘sanity check’ for the arbitrariness of the identified boundaries. If the nearly 50,000 detected boundaries were arbitrarily placed, then the pitch contours between them would have averaged out to produce flat mean curves. Instead, as we have shown, the average contour has distinct features that are consistent with what is known about pitch behavior. The observed declination in pitch therefore serves to validate the automatic identification of phrase boundaries.

### Phrases between consecutive boundaries exhibit a predictable syntactic structure

In this work, automatically identifying boundaries did not make use of syntactic information. Nevertheless, automatically obtained IUs do exhibit syntactic regularities (for positionally sensitive grammars see [61, 62]). First, the statistical trends unique to the first three positions of automatically identified phrases mirror those of manually detected IUs (Fig 5).

As shown in Table 2, frequent words in these positions are similar among the two methods and have distinct grammatical and discursive functions. For instance, the exclusion (to a large extent) of verbs from the first position and their frequent occupation of the second position is consistent with English being an SVO language. The high frequency of verbs in the third position is consistent with the prevalent practice in Spoken English to place coordinators and subordinators in unit-initial position. Coordination (typically “and” and less frequently “but” or “so”) is known to appear in spoken English three times more frequently than in correspondence and nine times more frequently than in academic writing [59]. Correspondingly, “and” was the most frequent word identified by either method of boundary detection. The frequency of the pronouns “I” and “you” in the first and second positions further indicates the known syntax of conversational language.

## Conclusion

In summary, we have presented a purely prosodic boundary detection that can efficiently tag phrase boundaries in spontaneous Spoken American English. It would seem natural, as a next step, to apply it to languages other than American English with similar typological features. Beyond the practical value of such boundary identification, analyzing large volumes of data in a timely fashion would enable to examine more elaborate models in the search for a dictionary of prosodic unit types and their functions. If so, a better understanding of the relations between conversational syntax and prosody may also be gained.
